# Editorial: The performance and adaptation of aquatic plants under global changes

**DOI:** 10.3389/fpls.2024.1380921

**Published:** 2024-03-08

**Authors:** Guixiang Yuan, Zhongqiang Li, Jianming Deng, Juan Pablo Pacheco, Hui Fu

**Affiliations:** ^1^ Hunan Provincial Key Laboratory of Rural Ecosystem Health in Dongting Lake Area, Ecology Department, College of Environment and Ecology, Hunan Agricultural University, Changsha, China; ^2^ Hubei Key Laboratory of Regional Development and Environmental Response, Faculty of Resource and Environment, Hubei University, Wuhan, China; ^3^ Taihu Laboratory for Lake Ecosystem Research, State Key Laboratory of Lake Science and Environment, Nanjing Institute of Geography and Limnology, Chinese Academy of Sciences, Nanjing, China; ^4^ Department Bioscience, Aarhus University, Silkeborg, Denmark

**Keywords:** macrophyte, microalgae, aquatic habitat, habitat fragmentation, adaptation strategies, genetic diversity, antibiotics degradation, eutrophic lake

Aquatic plants are primary producers in aquatic ecosystems and play a vital role in supporting ecological functions and services (Heino et al., 2015; Tasker et al., 2022). Global change, including intensified human activities and climate changes, drives serious habitat disturbances to aquatic plants, such as accelerated eutrophication, algal blooms, pollution by antibiotics, habitat fragmentation, extreme hydrological events as droughts and floods, warming and biological invasions (Robson et al., 2013; Hofstra et al., 2020; Yuan et al., 2023). These impacts in aquatic habitats often lead to a drastic plant loss, and may result in the expansion of harmful algal blooms, loss of plant vulnerable species, thus affecting the ecosystem’s capacity to support ecological functions and services (Lewington-Pearce et al., 2020; Tasker et al., 2022). Furthermore, these disruptive events may act synergistically to impact the performance of aquatic plants and may have a vicious cascade effect on the maintenance of aquatic plant populations and ecological functions.

Aquatic plants can improve their fitness in complex and changing habitats by adjusting physiological, phenotypic and genotypic traits, enzyme system activity and metabolite content (Xu et al., 2020; Yuan et al., 2023). However, the challenges faced by aquatic plants in terms of their performance and adaptation to disturbances caused by global change are still unclear. Consequently, further studies are urgently needed to assess the impact of global change disturbances on aquatic plant performance and adaptation strategies and the resulting cascading effects on the stability of freshwater ecosystems. This Research Topic brings together articles that explicitly address the ecological significance of aquatic plant performance and adaptation and that advance the understanding of the impacts of varying natural habitats under global change on aquatic plants and freshwater ecosystems.

Two papers focus on the ecological significance of performance responses of aquatic plants to varying habitats due to intensified human activities and/or global climate change. In a forum paper, Engloner et al. proposed that rivers with constant longitudinal connections by flowing water facilitated the high genetic variability for populations of a cosmopolitan plant *Ceratophyllum demersum* L. They suggest that the genetic diversity of aquatic plants with exclusive or dominant hydrochory could be severely affected by habitat fragmentation, for example due to climate change, subsequently impacting the adaptability of plant populations. Wang et al. showed that low-light stress, which occurred in eutrophic lakes, inhibited the growth of two common submerged macrophyte species (*Vallisneria natans* (Lour.) Hara and *Potamogeton maackianus* A. Bennett), and subsequently reduced zooplankton resource use efficiency by reducing zooplankton functional group richness and species diversity. They demonstrated a species-dependent influence of aquatic plants on crustacean zooplankton functional group richness, which are crucial for stabilizing clear water condition in shallow lakes.

Two papers report on the response of microalgae to the abiotic environment, e.g. increased antibiotic emissions and a diversified geographical environment. Huang et al. investigated the tolerance and removal efficiency of five freshwater microalgae and their microalgal consortia to the antibiotic, i.e., sulfamethoxazole. They found that the tolerance and sensitivity of the microalgae to sulfamethoxazole were species-specific, and the removal efficiency of sulfamethoxazole by the five microalgae ranges from 9% to 49%. Their study provides a new perspective for the selection of microalgal consortia to degrade antibiotics. Zeng et al. compared the phytoplankton communities in headwater streams with those in plain rivers. They demonstrated that for similar size watersheds, a lower spatio-temporal variability of the phytoplankton community was observed compared to plain rivers. The low nutrient content and strong hydrodynamics inhibited the growth of phytoplankton and reduced spatio-temporal variation, while at the same time contributing to great phytoplankton diversity in headwater streams.

The impacts of global change on terrestrial ecosystems have attracted considerable attention, but their impacts on aquatic ecosystems, in particular on the performance and adaptation of aquatic plants are still at a slow stage of development. Aquatic plants are widely distributed in natural water ecosystems around the world, and play a crucial role in the stability and continued functioning of ecosystems. Therefore, knowledge of aquatic plant performance is important for understanding how aquatic plants can successfully adapt to the constant and rapid changes occurring in natural habitats at a regional and global scales. We hope that the publication of this Research Topic will inspire other researchers to focus on this important issue in the coming years.

## Author contributions

GY: Writing – original draft. ZL: Writing – review & editing. JD: Writing – review & editing. JP: Writing – review & editing. HF: Writing – review & editing.
